# A Bayesian network perspective on neonatal pneumonia in pregnant women with diabetes mellitus

**DOI:** 10.1186/s12874-023-02070-9

**Published:** 2023-10-25

**Authors:** Yue Lin, Jia Shen Chen, Ni Zhong, Ao Zhang, Haiyan Pan

**Affiliations:** School of Public Health, Guangdong Medical University, Dongguan, 523808 China

**Keywords:** Bayesian networks, Neonatal pneumonia, Naive Bayes network, Tree Augmented Naive Bayes model, K-Dependence Bayesian Classifier

## Abstract

**Objective:**

To predict the influencing factors of neonatal pneumonia in pregnant women with diabetes mellitus using a Bayesian network model. By examining the intricate network connections between the numerous variables given by Bayesian networks (BN), this study aims to compare the prediction effect of the Bayesian network model and to analyze the influencing factors directly associated to neonatal pneumonia.

**Method:**

Through the structure learning algorithms of BN, Naive Bayesian (NB), Tree Augmented Naive Bayes (TAN), and k-Dependence Bayesian Classifier (KDB), complex networks connecting variables were presented and their predictive abilities were tested. The BN model and three machine learning models computed using the R bnlean package were also compared in the data set.

**Results:**

In constraint-based algorithms, three algorithms had different presentation DAGs. KDB had a better prediction effect than NB and TAN, and it achieved higher AUC compared with TAN. Among three machine learning modes, Support Vector Machine showed a accuracy rate of 91.04% and 67.88% of precision, which was lower than TAN (92.70%; 72.10%).

**Conclusion:**

KDB was applicable, and it can detect the dependencies between variables, identify more potential associations and track changes between variables and outcome.

**Supplementary Information:**

The online version contains supplementary material available at 10.1186/s12874-023-02070-9.

## Introduction

Gestational diabetes mellitus (GDM) is a common chronic disease of pregnancy that affects the health of tens of millions of women worldwide each year [1, 2]. During pregnancy, women experience disturbances in insulin secretion, leading to abnormal glucose metabolism, persistently elevated blood glucose levels and ultimately gestational diabetes mellitus, which is usually associated with adverse pregnancy outcomes [3, 4]. As well as affecting the mother’s own health, GDM can cause adverse pregnancy outcomes such as neonatal pneumonia [5, 6]. According to WHO and the Maternal Child Epidemiology Estimation (MCEE) group, a child will die from pneumonia every 43 s in 2020. Neonatal pneumonia is a serious threat to the health of newborn babies, and the disease can easily progress to respiratory failure or sepsis and other conditions, ultimately leading to neonatal death [7]. Therefore, it is very important to diagnose neonatal pneumonia in pregnant women with diabetes [8]. The current research focuses on the effect of neonatal ventilators on neonatal pneumonia, and the prediction models used are mainly logistic regression model [9].

How to classify efficiently and accurately has always been a problem in disease prediction. Common classification algorithms include Bayesian network [10], K-nearest neighbour algorithm [11] and decision tree [12]. Researchers take advantage of Bayesian network and machine learning methods to predict amyotrophic lateral sclerosis, and Bayesian network produced the best results [13]. However, by applying these models, it is difficult to reveal the potential information of neonatal pneumonia of gestational diabetes, which is a complex disease affected by multiple factors. Although many scholars at home and abroad have studied the effects of gestational diabetes on maternal and infant perinatal outcomes, the method of Bayesian network (BN) model has not been applied to gestational diabetes complicated with neonatal pneumonia.

Bayesian network is an effective hotspot method that has been applied to disease data mining research in recent years. Bayesian network has several advantages that make it a promising tool for these purposes. It is an uncertain causal relationship model that organically combines directed acyclic graph with probability theory, which represents directly and intuitively. Integrating data into the model in the form of conditional probability can deal with various uncertainties and incomplete information. In addition, the Bayesian network can predict the effectiveness of an intervention strategy by introducing new evidence, which is an important and unique advantage over other methods [14].

As it is shown [13, 15], BN is very useful for prediction and diagnosis, which is very important in disease interventions because they are usually expensive and their effects can only be observed in the long term. BN has the properties to be very useful in predicting the effectiveness of different strategies and selecting the best among them. Currently there are many advances in Bayesian classifiers such as Naive Bayes (NB), TAN and so on. Naive Bayes (NB) is one of the simplest and most efficient BNs due to the independence of hypothetical features. The independence hypothesis between features is usually not true, so relaxing the independence hypothesis and expanding the dependency of the Bayesian network has become the main improvement of Naive Bayes, among which the more successful algorithms are Tree-Augmented Naive Bayes (TAN) and k-Dependence Bayesian Classifier (KDB) [16].

In this study, three Bayesian network models, namely Naive Bayes, Tree Augmented Naive Bayes and k-Dependence Bayesian Classifier are used to predict the risk of neonatal pneumonia in pregnancy diabetes. The prediction accuracy and recall rate are compared internally and externally with three machine learning models such as Decision Tree (DT), Random Forest (RF) and Support Vector Machine (SVM). At the same time, Bayesian networks known as Directed Acyclic Graphs (DAGs) are analysed to find the advantages, disadvantages and scope of Bayesian network models.

## Materials and methods

### Data

A total of 2008 pregnant women with diabetes who gave birth at Shunde Women and Children’s Hospital of Guangdong Medical University between June 2019 and June 2021 were included: 305 pregnant mothers whose babies had neonatal pneumonia (case group), and 1703 who did not have neonatal pneumonia (control group). Written informed consent was obtained from all recruited participants or their legal guardians, and this study was approved by the Ethics Committee of Foshan Women’s and Children’s Hospital of Guangdong Medical University (SDFYMC001).

### Univariate analysis of variables

The chi-squared test was performed on the categorical variables. The 13 variables included in the model were age (age), two-hour postprandial glucose (pbg), parity (p), gestational hypertension (hdop), preterm birth (ptb), preterm rupture of membranes (prom), macrosomia (ms), neonatal respiratory distress syndrome (nrds), neonatal jaundice (nnj), postpartum haemorrhage (pph), neonatal asphyxia (na) and neonatal growth restriction (ngr). Mann–Whitney U test was performed on the rank variables and 4 variables were included in the model: number of pregnancies (g), amniotic fluid volume (afv), amniotic fluid cleanliness (afc) and C-reactive protein (crp).

### Software and programs

**Fig. 1:**
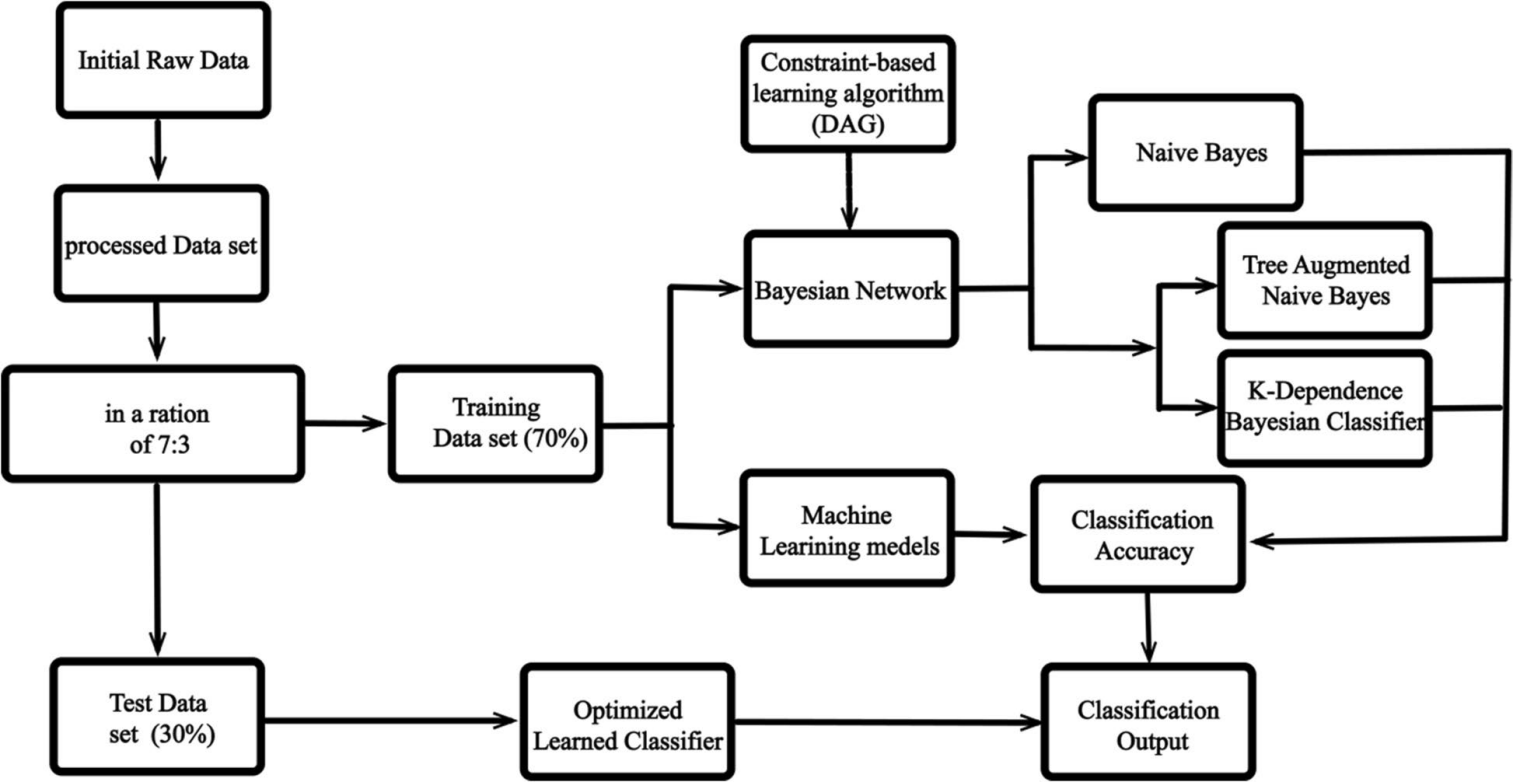
A schematic diagram of methodology

In the study, our first step is to perform a BN analysis to build a causal graphical model in the form of a directed acyclic graph (DAG), which represents the relationships between all the variables of interest [17]. Bayesian networks can be constructed using structure learning algorithms, which can be categorised into two main groups: constraint-based and score-based methods. In this article, three types of constraint-based learning algorithms were used with the R package bnlearn [18]. In order to ensure that the resulting network was stable, we performed bootstrapping by extracting 1000 samples with replacement, computing a network for each sample, and then averaging them to obtain the resulting network. The study then performed an analysis of the intercorrelation between the feature variables before selecting an appropriate Bayesian method. As expected, Naive Bayes (NB), Tree-augmented Naive Bayes (TAN) and K-Dependence Bayesian Classifier (KDB) were then selected to build the BN. However, NB is used in the study for parameter learning due to its hypothesis [19, 20]. At the same time, its performance is compared with three types of machine learning methods, Random Forest, SVM and DT. The Netica software package developed by Norsys Software Corporation was used to perform TAN [21, 22]. A schematic diagram of methodology is shown in Fig. 1:

## Bayesian network

### Bayesian network (BN)

.Bayesian networks (BNs), also known as probabilistic directed acyclic graphs (DAGs), are directed networks accompanied by probabilistic links between edges. A graph is a DAG if all the links (edges) have directions, but none of the nodes go directly to itself or through a path to itself (a circle) [23]. Bayesian networks are able to connect probability distributions on a finite set of random variables. Directed edges represent statistical or causal dependencies between variables [24]. For example, given an edge X → Y, X is the parent node of Y and Y is the child node. Each node, e.g. Xi, has a conditional probability distribution that quantifies the effect of the parent on the child node. In general, the joint conditional probability distribution of any combination of random variables is simplified to formula (1).

1 $$P\left(x_1,x_2,x_n\right)={\textstyle\prod_{i=1}^n}P\left(x_i\left|Parents\left(x_i\right)\right.\right)$$

The parent nodes of a particular node are its immediate predecessors within the network. These parent nodes of a particular node are its immediate predecessors within the network. These parent nodes are the variables that directly influence the associated node. In Bayesian networks, the term “parent node set” refers to the aggregation of all the parent nodes that influence a particular node. A node has several parents, which together form a set of parents. Ancestor nodes, on the other hand, include all parent nodes, their parent nodes, and so on, forming a lineage of dependencies tracing back to the most distant nodes in the network. The concept of a “Markov Blanket” is the minimal set of nodes that contains all the information necessary to specify the conditional probability distribution of a given node, given its parent and child nodes. The high dimensionality of the data has led to the development of several learning algorithms that focus on reducing computational complexity while still learning the correct network. On the one hand, among several structure learning algorithms [25], constraint-based learning algorithms consist of growth-shrink, fast.iamb and mmpc etc. and it provides a free implementation of some of these structure learning algorithms along with the conditional independence tests and network scores used to construct the Bayesian network. The resulting models are often interpreted as causal models.

### Naive Bayes

Naive Bayesian is a simple, stable, easy-to-implement Bayesian algorithm with better classification efficiency based on the assumption that each feature condition is independent of each other [19]. Its algorithm is as follows:

① Supposing that $x=\left\{{a}_{1},{a}_{2},\cdot \cdot \cdot ,{a}_{m}\right\}$ is an item to be classified, and each *a*_*i*_ is a characteristic attribute of x;
② A set of categories $C=\left\{{\mathrm{y}}_{1}{,\mathrm{y}}_{2},\cdot \cdot \cdot {,\mathrm{y}}_{\mathrm{n}}\right\};$ 
③ Calculate the conditional probability of each feature, namely: 
  $$P\left({y}_{1}\left|x\right.\right),P\left({y}_{2}\left|x\right.\right),\cdot \cdot \cdot , P\left({y}_{n}\left|x\right.\right)$$;
④ Take the maximum conditional probability: 
  $$P\left({y}_{k}\left|x\right.\right)=max\left\{P\left({y}_{1}\left|x\right.\right),P\left({y}_{2}\left|x\right.\right),\cdot \cdot \cdot , P\left({y}_{n}\left|x\right.\right)\right\}$$; then $$x\in {y}_{k}$$.

The calculation process of conditional probability is as follows:

① Establish a sample data set as a training sample set.
② Calculate the conditional probability of each eigenvalue under each category 
$$P\left({a}_{i}\left|{y}_{j}\right.\right)\left(1\le i\le m,1\le j\le n\right)$$
③ Assuming that all attributes are independent of each other, then according to Bayes’ theorem we have drawn formula (2):

2 $$P\left({y}_{1}\left|x\right.\right)=\frac{P\left(x\left|{y}_{i}\right.\right)P\left({y}_{i}\right)}{P(x)}$$

Since the denominator is a constant, the largest numerator is needed taken, adding to that each attribute is independent of each other, there are shown in formula (3):

3 $$P\left(x\left|y_i\right.\right)P\left(y_i\right)=P\left(a_1\left|y_i\right.\right)P\left(a_2\left|y_i\right.\right)\cdot\cdot\cdot P\left(a_m\left|y_i\right.\right)=P\left(y_i\right){\textstyle\prod_{j=1}^m}P\left(a_j\left|y_i\right.\right)$$

**Fig. 2:**
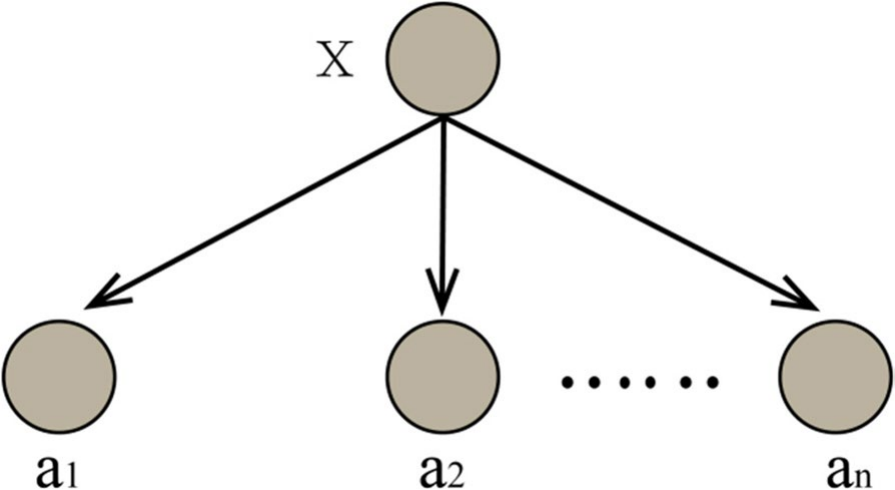
The structure of NB

When attribute events are independent of each other, the accuracy of Naive Bayesian classification is very good. Figure 2 shows the structure of NB. In reality, each feature variable is often not conditionally independent, but has a dependency relationship, which limits Naive Bayesian classification ability.

### Tree Augmented Naive Bayes (TAN)

**Fig. 3:**
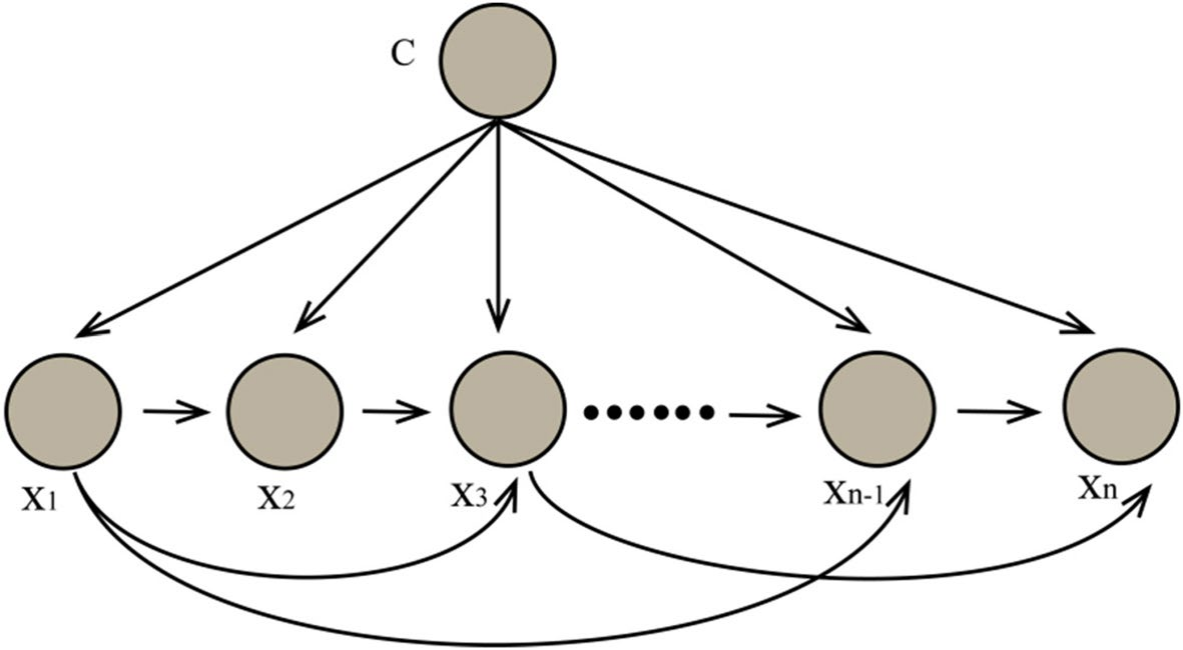
The structure of TAN

Tree Augmented Naive Bayes (TAN) is a type of Bayesian network that is an improvement on NB. It assumes that the relationships between attribute variables conform to a qualified tree structure. The basic concept is to break the independence assumption of NB and allow dependencies between categorical variables, but a categorical variable is allowed to have a dependency with at most one other categorical variable. This dependency is represented by a tree structure [26]. Figure 3 shows the structure of TAN.

Construction of Tree Augmented Naive Bayes Network (TAN) contains two parts, structure learning and parameter learning:

① *X*_*j*_ provides information for *X*_*i*_ when C is known, represented by mutual information. Calculate $X_{i}$ of attribute C according to the data set of training set.

4 $$I\left(X_i,X_j\left|C\right.\right)={\textstyle\sum_{x_i,x_j,c}}P\left(x_i,x_j,c\right)\times log\frac{P\left(x_i,x_j\left|c\right.\right)}{P\left(x_i\left|c\right.\right)P\left(x_j\left|c\right.\right)}$$

In formula (4), $P\left({x}_{i},{x}_{j},c\right)$ represents the probability of occurrence of features ${x}_{i}$、${x}_{j}$, and category $c$, and logarithms are used to avoid numerical issues. The formula $\mathrm{log}\left(P\left({x}_{i},{x}_{j}\left|c\right.\right)/P\left({x}_{i}\left|c\right.\right)P\left({x}_{j}\left|c\right.\right)\right)$ is used to calculate the mutual information between features ${x}_{i}$ and ${x}_{j}$, which measures their dependence. If features ${x}_{i}$ and ${x}_{j}$ are independent, then $P\left({x}_{i},{x}_{j}\left|c\right.\right)$ equals $P\left({x}_{i}\left|c\right.\right)$×$P\left({x}_{j}\left|c\right.\right)$, and the mutual information is 0. If features ${x}_{i}$ and ${x}_{j}$ are dependent, then $P\left({x}_{i},{x}_{j}\left|c\right.\right)$ is not equal to $P\left({x}_{i}\left|c\right.\right)$×$P\left({x}_{j}\left|c\right.\right)$, and the mutual information is greater than 0.

② The maximum weight span tree is established by using the conditional mutual information of the node pair as the weight of the edge. First, sort the edges according to their weights, and then select the edges in order. After each edge is selected, check whether the tree contains cycles. If it contains cycles, delete the edge and select the next edge. The resulting tree is the maximum weight span tree. Finally, take a node as the starting point, and the direction of leaving the node is used as the direction of the edge in the tree;
③ Add class node as parent node for all attribute nodes;
④ Calculate the joint probability of each classification node to get the TAN classifier.

### K-Dependence Bayesian Classifier(KDB)

**Fig. 4:**
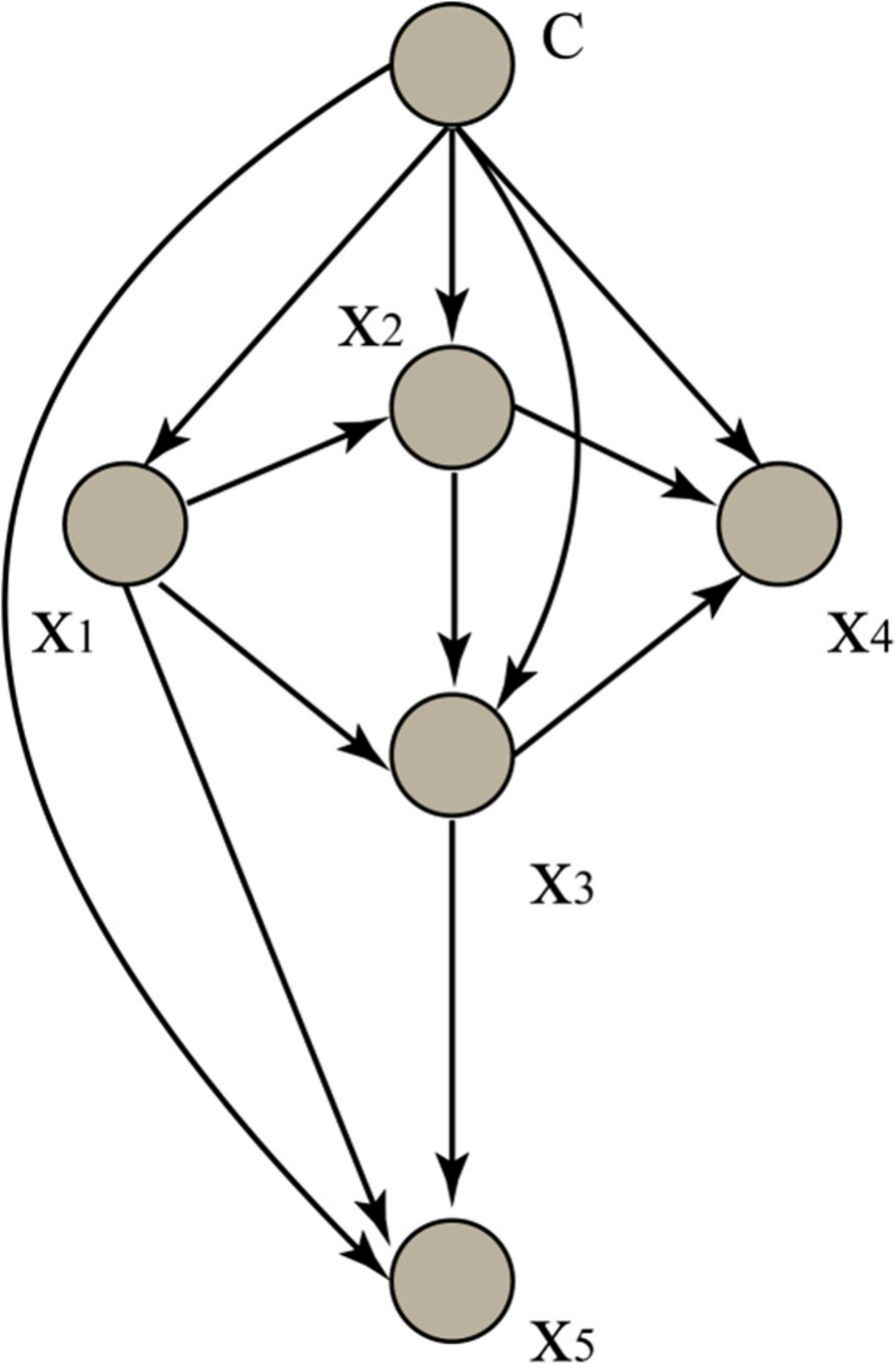
The structure of KDB (k = 2)

KDB is also an improvement on the assumption of conditional independence of the Naive Bayes [27, 28]. Compared with TAN and NB, KDB allows high-order conditional dependencies between attributes, further alleviating dependencies between attributes. KDB sort the attributes based on MI $\left(Xi;Y\right)$, then add them to the network in turn, and according to CMI $\left(Xi;{X}_{i}\left|Y\right.\right)$ selects K attributes as its parent node. As a result, KDB can make better use of the information in the dataset and can perform better. KDB further frees up the limitations of TAN, allowing an attribute to have up to k attributes as its parent node. In general, k(1≦k≦n-1) is determined before building the model, and the structure diagram is shown in Fig. 4, k = 2. However, k can be freely adjusted, which makes KDB extremely flexible and malleable. Assume that the attribute order is {X_1_,…, X_n_}, by comparing MI, Xi will choose min(i − 1,k) features with the highest CMI values from the first i − 1 candidates. The joint probability for KDB proves to be formula (5):

5 $${\boldsymbol P}_{KDB}(\boldsymbol x,\;c)=P(c){\textstyle\prod_{i=1}^n}P(x_i\vert c,\pi_{x_i})$$

### Model evaluation

Firstly, this study uses the changes of node parameter values in two Bayesian network models to evaluate their applicable conditions. Secondly, each sample can be divided into four cases: true positive (TP), false positive (FP), true negative (TN), and false negative (FN) according to the combination of its real category and the prediction category of each model, so that TP, FP, TN, FN represent their corresponding sample cases, and the “confusion matrix” of the classification results can be obtained, as shown in the Table 1.

**Table 1:** Confusion matrix

	True value	
	Positive	Negative
Predicted value		
Positive	TP	FN
Negative	FP	TN

The authenticity of each model was evaluated by evaluation indicators such as accuracy, sensitivity, specificity, positive predictive value, and negative predictive value. Among it, accuracy = (TP + TN)/(TP + FN + FP + TN), sensitivity = TP/(TP + FN), specificity = TN/(FP + TN), positive predictive value = FP/(TP + FP) and negative predictive value = FN/(TN + FN).

## Results

### The results of the constraint-based learning algorithm

**Fig. 5:**
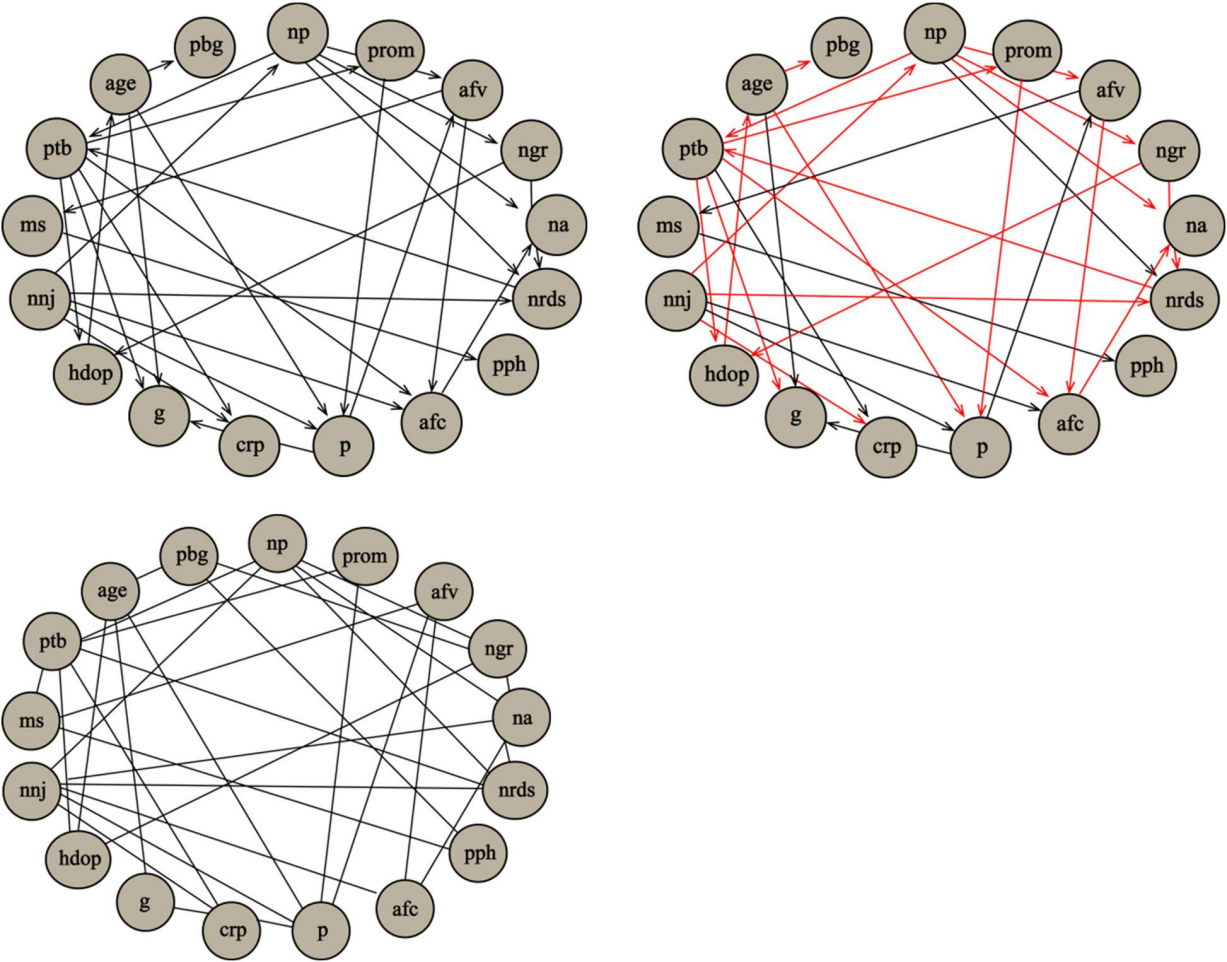
The Growth-Shrink network structure (top left) and the network structures learned by fast.iamb (top right), mmpc (bottom left)

Constraint-based learning algorithm. Even while all the algorithms produce network structures that are remarkably similar and agree on the arc direction (as shown in Fig. 5), there are notable differences: The graph’s layout shows that mechanics and variables are crucial to the overall assessment of the test, in all models the analysis and statistics scores are conditionally independent of each other. Fast.iamb has 23/14 of directed/undirected arcs while Growth-Shrink has 29/8 of directed/undirected arcs; mmpc learns the underlying structure of the Bayesian network with all the arcs undirected. The red line depicts the variation between fast.iamb and growth-shrink. However, demonstrating the real connections between variables is difficult.

### Building a Naive Bayesian network model

Multivariate correlation analysis was conducted on all the varibales. Then we developed separate NB and evaluate the contributions of ptb, crp, nrds, ngr, afv, and prom in neonatal pneumonia. Factual or reference status is introduced to show the counterfactual condition with or without a patient-specific factor regarding its impact on neonatal pneumonia (node “np”) respectively. In the complex correlation analysis model (np ~ ptb + crp + nrds + ngr + afv + prom, *R*^2^≧0.6), indicating the goodness of the model as well as np [13]. The relationship among patient-specific factors and neonatal pneumonia in an NB is fixed, and among them nodes represent these factors and neonatal pneumonia, directed arcs denote dependent relationship between each factor and the stage. Although NB is based on the assumption of independence among all features, we build a NB in this dataset by R. When the test set samples (30% of the data) are imported to verify the prediction performance of the model, Naive Bayesian has accuracy rate of 92.20% and achieves the performance with ROC of 94.64%. The results are shown in Table 2. Relatively simple structure may result from underfitting rather than overfitting may help to improve the classification performance of learning algorithm.

**Table 2:** Prediction performance of Naive Bayesian

	Prediction					
	Neonatal pneumonia					
Measured	sick	normal	recall rate(%)	precision rate(%)	accuracy rate(%)	F1-score(%)
Neonatal pneumonia						
sick	61	25	73.4	70.9	92.20	72.12
normal	22	495				

### Build Tree Augmented Naive Bayes (TAN)

**Fig. 6:**
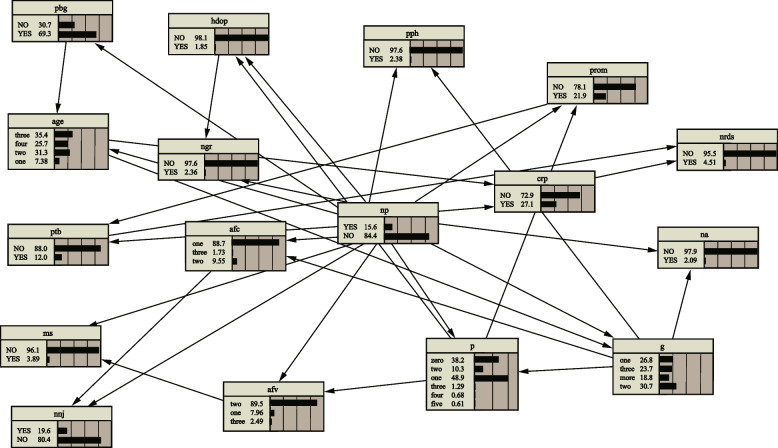
Tree Augmented Naive Bayes (TAN)

The data was divided into the training set and the test set in a ratio of 7:3 and the Tree Augmented Naive Bayes was learned and tested on Netica software, as shown in Fig. 6.

**Fig. 7:**
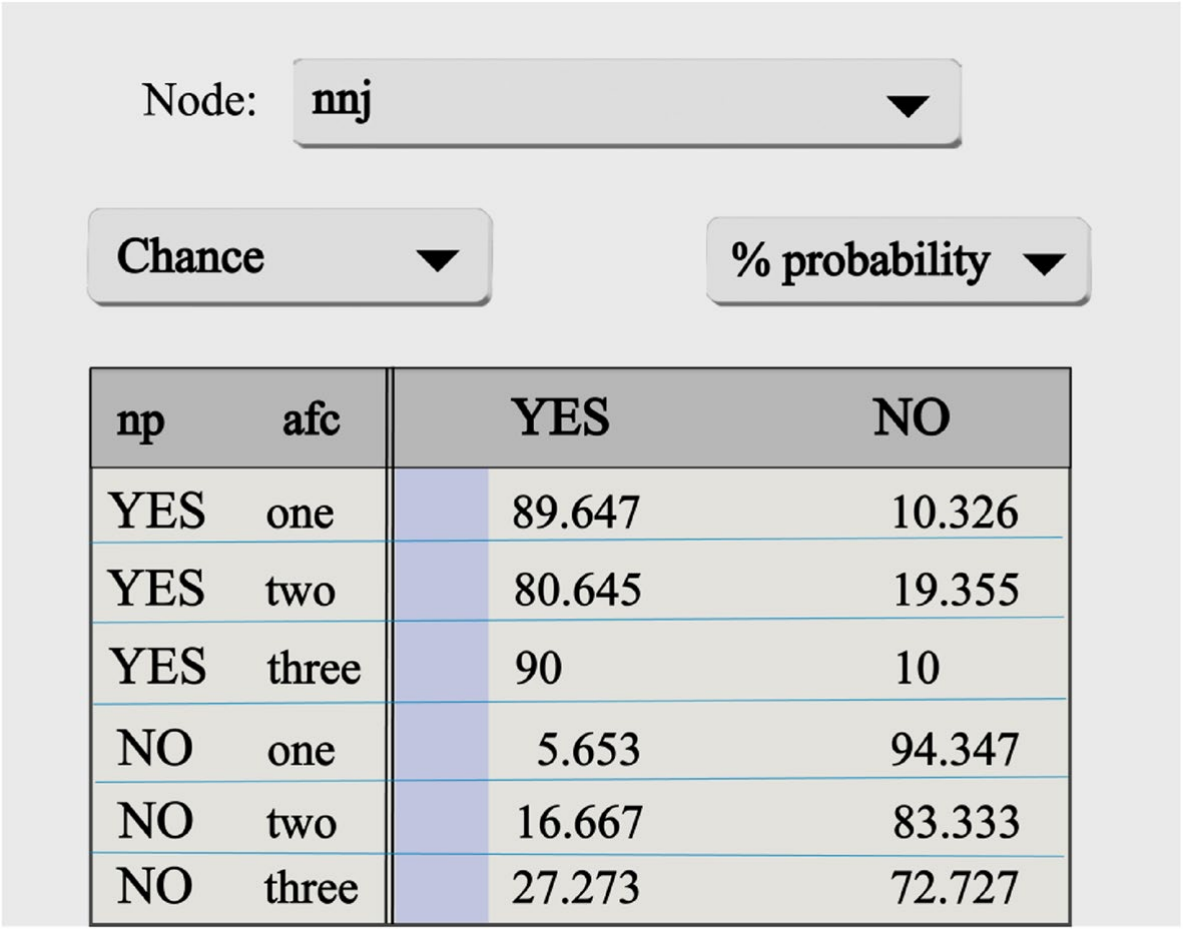
Conditional probabilities of neonatal jaundice (nnj)

As we can see, TAN allows a dependency between an attribute variable, so more information can be obtained by looking at the conditional probability table of the node. Take the example of neonatal jaundice (nnj), as shown in Fig. 7. The data in Fig. 6 represent the corresponding changes in the parameters of neonatal jaundice (nnj) under different conditions of neonatal pneumonia (np) and amniotic fluid cleanliness (afc) in the two parent nodes connected to it. If a patient has neonatal pneumonia (np) and amniotic fluid cleanliness (afc) is the first level, the probability of occurrence of neonatal jaundice (nnj) is 89.7% and the probability of non-occurrence is 10. 3%; if the patient does not develop neonatal pneumonia (np), but amniotic fluid cleanliness (afc) is the third level, the probability of neonatal jaundice is 27.27% and the probability of non-occurrence is 72.72%. Thus, neonates from neonatal jaundice populations are more likely to have amniotic fluid cleanliness and neonatal pneumonia.

The test set are imported into the model in the TAN to verify the prediction performance. The results are shown in Table 3. TAN has accuracy rate of 92.7%, a recall rate of 68.6% and a precision rate of 74.68%. Besides, TAN achieves the performance with ROC of 93.78%.

**Table 3:** Prediction performance of TAN

	Prediction					
	Neonatal pneumonia					
Measured	sick	normal	recall rate(%)	precision rate(%)	accuracy rate (%)	F1-score(%)
Neonatal pneumonia						
sick	62	24	75.60	72.10	92.70	73.81
normal	20	497				

### Build K-Dependence Bayesian Classifier (KDB)

**Fig. 8:**
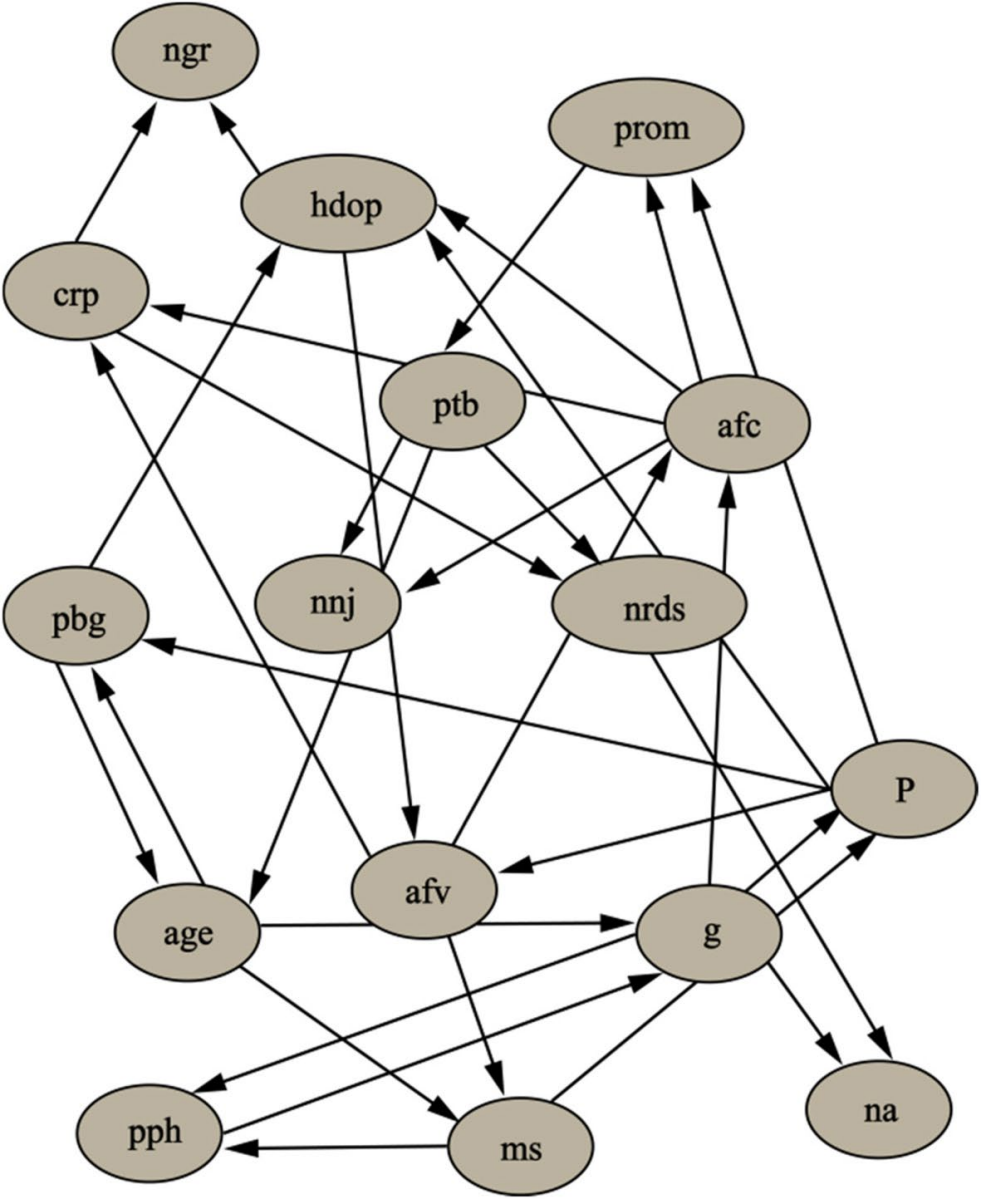
Conditional dependencies between attributes are shown (KDB)

The algorithms KDB were developed in C +  + using the NetBeans IDE compiler + GCC. The data was divided into the training set and the test set in a ratio of 7:3. KDB treats training set as a target and build general BN. When k = 2, KDB can represent 0 + 1 + 2⋯ + 2 = 33 conditional dependencies, while TAN only needs to represent 16 conditional dependencies, shown in Fig. 8. In this data, KDB (0.95 ± 0.09) achieves higher AUC compared with TAN (0.95 ± 0.10). As a result, the KDB does fit the testing instance much better than TAN.

### The comparison of modes performance

The data were split 7:3 into training and test sets. Data analysis was performed in the R environment (R Foundation for Statistical Computing, Beagle Scouts, version 4.3.1., https://cran.r-project.org/src/base/R-4, using the KlaR, rRpart, randonForest, pROC and e1071 libraries). Three machine learning methods were used to construct the prediction model for neonatal pneumonia and the predictive performance of these three models is compared. Table 4 describes the performance of the prediction models considered. The Support Vector Machine ( AUC, 0.957) achieved the accuracy rate of 91.04%. However, there is no difference between Decision Tree and Random Forest (AUC, 0.951).

**Table 4:** Model performance

Model	Accuracy(%)	Precision(%)	Recall(%)	F1-score(%)
Decision Tree	90.71	69.51	64.77	65.59
Support Vector Machine	91.04	67.88	90.29	77.49
Random Forest	90.71	75.27	67.96	71.43

## Discussion

In this study, we first applied structure learning algorithms and created a directed acyclic graph (DAG) for neonatal pneumonia. Although it appeared that neonatal pneumonia was more likely to affect linked variables, it provided a new way to understand the aetiology and generate hypotheses about potential causal symptom structures and identify factors that may bridge neonatal pneumonia. Secondly, the influence of the pregnant population on the outcome of neonatal pneumonia was investigated. The naive Bayesian learnt that preterm birth, C-reactive protein, neonatal growth restriction, neonatal respiratory distress syndrome, amniotic fluid volume and premature rupture of membranes have a greater impact on the outcome of neonatal pneumonia. As a result, it has a predictive performance of 92.20% for neonatal pneumonia. On the other hand, Tree Augmented Naive Bayes found that age was associated with pregnancies and the index of C-reactive protein affecting neonatal pneumonia. At the same time, pregnancies affect parity and amniotic fluid volume, circularly increasing the risk of macrosomia. The performance of KDB appears to be slightly higher than that of TAN, but the difference is very small. In addition, we were interested in building machine learning models to predict the occurrence of neonatal pneumonia and make a comparison with Bayesian network models. Although the support vector machine showed a better area under the ROC curve, it is still lower than the Bayesian network model [29].

As for the factors of neonatal pneumonia, studies have reported that the blood glucose level of pregnant women can affect fetal development, leading to adverse pregnancy outcomes such as macrosomia and premature rupture of membranes [30–33], as shown in KDB (pbg → age → ms). It can be seen that if the mother continues to have high blood glucose levels, this will affect the health of the newborn. This is because high blood glucose levels can reduce leukocyte phagocytosis and chemotaxis in pregnant women, which increases the risk of urinary tract and reproductive tract infections, thereby increasing the risk of premature rupture of membranes (age → pbg → prom) in the Bayesian network of KDB [34]. Studies have shown that in the gestational diabetes population, the rate of neonatal pneumonia is lower in the well-controlled group than in the poorly controlled group [29]. Specifically, pregnant women and fetuses have a special relationship in which any physical changes in the mother will affect the fetus and even affect its future growth and development (pbg → hdop → ngr in KDB). Neonatal gestational diabetes affects neonatal lung development, neonatal lung maturation and associated lung diseases (np → pbg; np → hdop in TAN), with a high incidence of neonatal pneumonia, neonatal respiratory distress syndrome and bronchopulmonary dysplasia in gestational diabetes patients [34, 35].

Compared with the traditional regression model, the Bayesian network model can handle large sample data. While compared with machine learning with poor interpretability [36], the Bayesian networks built on the coefficients reveal some patterns of disease variables, which have the potential to help diagnose the disease [23].The Bayesian network returns a DAG that identifies the direction of prediction and potential causal influence among factors in the absence of a randomised controlled experiment, but cross-sectional data cannot confirm to the true situation. Furthermore, in a DAG, activation flows in only one direction, never returning to the node of origin. Therefore, there are no important variables influencing associations between risk factors that have been omitted from the DAG. Furthermore, such instability in the direction of the edge may suggest bidirectionality of influence, considering that in 51% of the bootstrapped samples the edge points from factor X to factor Y, and in 49% of the bootstrapped samples from factor Y to factor X [27]. In research, TAN can observe the dependencies between attribute variables and reveal the strength of their dependencies, although the naive Bayesian has higher restrictions that require variables to be independent of each other.

When it comes to K-dependence Bayesian classifiers, it has been proposed to mine dependency relationships from the data. For KDB, all features are indiscriminately conditionally dependent on at most k parent features, even if the conditional dependencies are very weak. KDB provides the “average network” to express significant dependencies, so it cannot apply to all cases. At the same time, KDB cannot accurately describe the dependency relationships in different patient records [37]. However, KDB has satisfactory classification accuracy when dealing with large samples. In addition, KDB uses a single parameter k to determine the number of parents for each feature, thus controlling the complexity of the structure [38]. In the study, the full network structure with 16 attributes is too complex (32 arcs or conditional dependencies) to explain, so we only select a substructure to clarify. The disadvantage of KDB in terms of extensibility is obvious. As shown in Fig. 8, the value afv (amniotic fluid volume) is a precondition of the value afc (amniotic fluid cleanliness). If they appear as co-parents of some other attribute, e.g. crp (C-reactive protein), the conditional probability P(crp|afv, afc, np) will approximate the estimate of P(crp|afc, y) and afv (amniotic fluid volume) cannot provide valuable information on amniotic fluid cleanliness.

The limitation of this study is that only 16 variables were included into the model, and many variables that were not statistically significant were excluded. In fact, many influencing factors must be considered in the application. At the same time, these variables may have collinearity problems, and subsequent research can also consider combining principal component analysis or factor analysis to reduce the dimensionality of variables. Some continuous variables are discretized, which leads to the waste of data information. In this paper, constraint-based learning algrithms may be useful to compare different network structures for the same data, to verify the goodness of fit of the learned network with respect to a particular score function where our future work will focus on. Since the structures is not evident in the Max–Min Hill-Climbing (MMHC) hybrid algorithm, and some hybrid algorithms such as MMPC-Tabu, Fast.iamb-Tabu and Inter.iamb-Tabu. However, we should look at it in a dialectical way where the ML models in another set of datasets is to test model accuracies, whether it is consistent or not relies on one dataset [38]. Our next step is to assess their performance and make a comparison between the widely used Bayesian network algorithm on more generated datasets, and explore a new hybrid Bayesian network method.

### Supplementary Information

**Additional file 1: Figure S1.** Bayesian network with MMHC^[1]^. **Figure S2.** Bayesian network with Fast.iamb-Tabu^[1]^. **Figure S3.** Bayesian network with Inter.iamb-Tabu^[1]^. **Figure S4.** Bayesian network with MMHC.Tabu^[1]^. **Figure S5.** Bayesian network (hill climbing, directed acyclic graph)^[2]^. **Figure S6.** Bayesian network (Scutari & Nagarajan’s (2013) method)^[2-4]^.

## Data Availability

All data generated or analysed during this study are included in this published article.
