# Supplementary material for: A Bayesian network perspective on neonatal pneumonia in pregnant women with diabetes mellitus
Source: BMC Med Res Methodol. 2023 Oct 25;23:249. doi: 10.1186/s12874-023-02070-9 (PMC10601254; doi:10.1186/s12874-023-02070-9)
Supplement: Supplementary file 1 — Additional file 1: Figure S1. Bayesian network with MMHC[1]. Figure S2. Bayesian network with Fast.iamb-Tabu[1]. Figure S3. Bayesian network with Inter.iamb-Tabu[1]. Figure S4. Bayesian network with MMHC.Tabu[1]. Figure S5. Bayesian network (hill climbing, directed acyclic graph)[2]. Figure S6. Bayesian network (Scutari & Nagarajan’s (2013) method)[2-4]. [file 12874_2023_2070_MOESM1_ESM.pdf]

Hybrid Algorithms of Bayesian Network[1]:

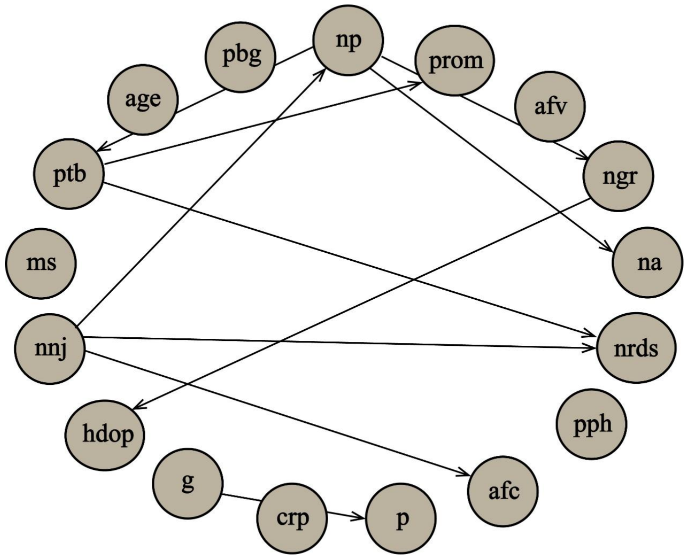

Figure S1

Bayesian network with MMHC[1]

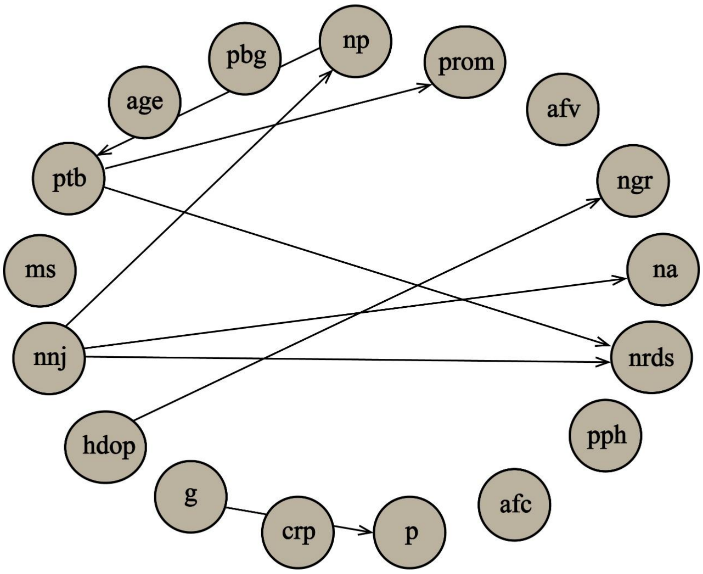

Figure S2

Bayesian network with Fast.iamb-Tabu[1]

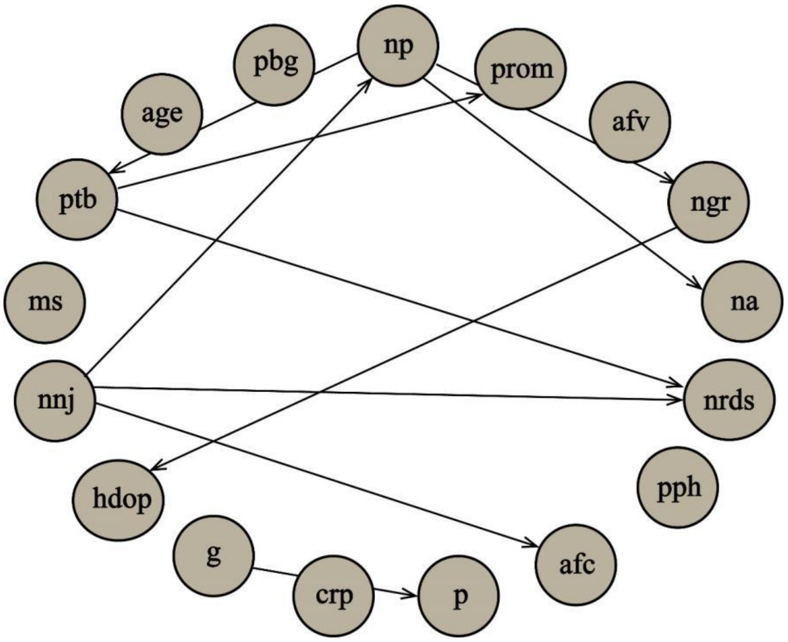

Figure S3

Bayesian network with Inter.iamb-Tabu[1]

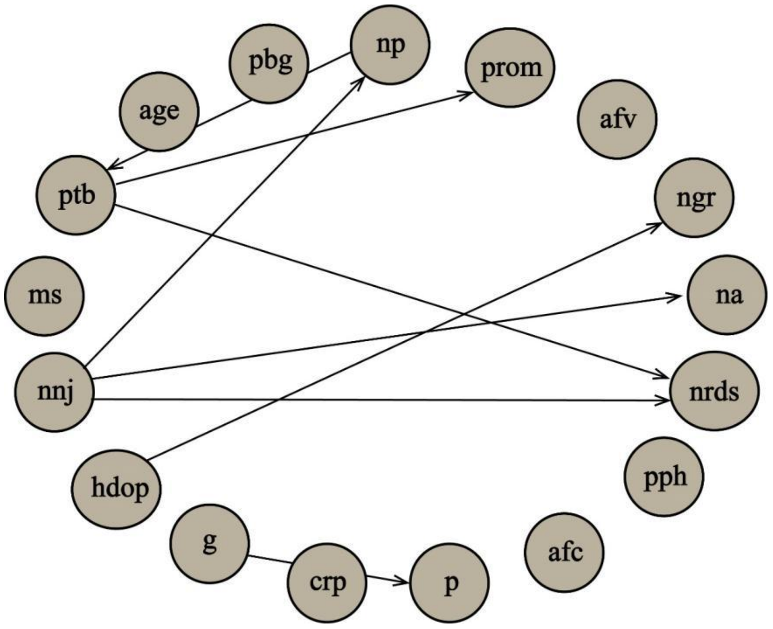

Figure S4

Bayesian network with MMHC.Tabu[1]

Directed Acyclic Graph (DAGs)of Bayesian network[2-4]:

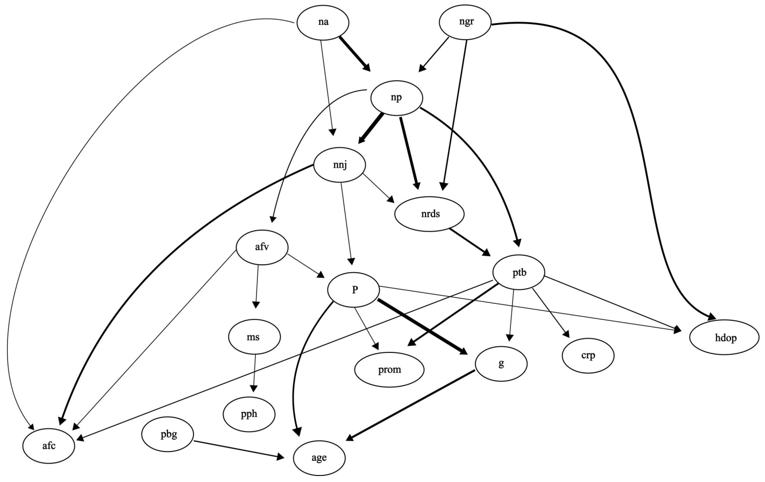

Figure S5

Bayesian network (hill climbing, directed acyclic graph)[2]

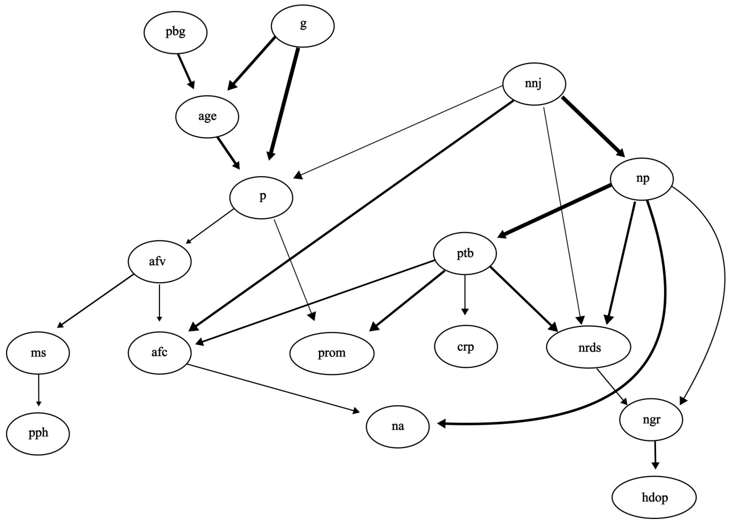

Figure S6

Bayesian network (Scutari & Nagarajan's (2013) method)[2-4]

Reference

[1] Wang X, Pan J, Ren Z, et al. Application of a novel hybrid algorithm of Bayesian network in the study of hyperlipidemia related factors: a cross-sectional study. BMC Public Health. 2021;21(1):1375. Published 2021 Jul 12.

[2] Scutari M (2010). Learning Bayesian networks with the BNLEARN package. Journal of Statistical Software 35, 1–22.

[3] Scutari M, Denis J-P (2015). Bayesian Networks: With Examples in R. CRC Press: Boca Raton, FL.

[4] Scutari M, Nagarajan R (2013). Identifying significant edges in graphical models of molecular networks. Artificial Intelligence in Medicine 57, 207–217.
